# Evaluation of different mathematical models and different b-value ranges of diffusion-weighted imaging in peripheral zone prostate cancer detection using b-value up to 4500 s/mm^2^

**DOI:** 10.1371/journal.pone.0172127

**Published:** 2017-02-15

**Authors:** Zhaoyan Feng, Xiangde Min, Daniel J. A. Margolis, Caohui Duan, Yuping Chen, Vivek Kumar Sah, Nabin Chaudhary, Basen Li, Zan Ke, Peipei Zhang, Liang Wang

**Affiliations:** 1 Department of Radiology, Tongji Hospital, Tongji Medical College, Huazhong University of Science and Technology, Wuhan, Hubei, China; 2 Department of Radiology, David Geffen School of Medicine at UCLA, Ronald Reagan UCLA Medical Center, Los Angeles, California, United States of America; 3 Key Laboratory of Magnetic Resonance in Biological Systems, State Key Laboratory of Magnetic Resonance and Atomic and Molecular Physics, National Center for Magnetic Resonance in Wuhan, Wuhan Institute of Physics and Mathematics, Chinese Academy of Sciences, Wuhan, Hubei, China; 4 Centers for Biomedical Engineering, University of Science and Technology of China, Hefei, Anhui, China; Eberhard Karls University, GERMANY

## Abstract

**Objectives:**

To evaluate the diagnostic performance of different mathematical models and different b-value ranges of diffusion-weighted imaging (DWI) in peripheral zone prostate cancer (PZ PCa) detection.

**Methods:**

Fifty-six patients with histologically proven PZ PCa who underwent DWI-magnetic resonance imaging (MRI) using 21 b-values (0–4500 s/mm^2^) were included. The mean signal intensities of the regions of interest (ROIs) placed in benign PZs and cancerous tissues on DWI images were fitted using mono-exponential, bi-exponential, stretched-exponential, and kurtosis models. The b-values were divided into four ranges: 0–1000, 0–2000, 0–3200, and 0–4500 s/mm^2^, grouped as A, B, C, and D, respectively. ADC, <D>, D*, f, DDC, α, D_app_, and K_app_ were estimated for each group. The adjusted coefficient of determination (R^2^) was calculated to measure goodness-of-fit. Receiver operating characteristic curve analysis was performed to evaluate the diagnostic performance of the parameters.

**Results:**

All parameters except D* showed significant differences between cancerous tissues and benign PZs in each group. The area under the curve values (AUCs) of ADC were comparable in groups C and D (*p* = 0.980) and were significantly higher than those in groups A and B (*p*< 0.05 for all). The AUCs of ADC and K_app_ in groups B and C were similar (*p* = 0.07 and *p* = 0.954), and were significantly higher than the other parameters (*p*< 0.001 for all). The AUCs of ADC in group D was slightly higher than K_app_ (*p* = 0.002), and both were significantly higher than the other parameters (*p*< 0.001 for all).

**Conclusions:**

ADC derived from conventional mono-exponential high b-value (3200 s/mm^2^) models is an optimal parameter for PZ PCa detection.

## Introduction

Magnetic resonance imaging (MRI) is considered the best imaging technique for the detection, staging and surveillance of prostate cancer (PCa) because of its ability to provide functional and anatomic information [1–3]. Diffusion-weighted imaging (DWI) is a powerful and dominant component of MRI in peripheral zone (PZ) PCa detection[4].

In traditional diffusion theory, a mono-exponential model, based on the assumption of homogeneous Gaussian diffusion, has been adopted for diffusion analysis in most clinical studies [5, 6]. However, molecular diffusion in biological tissues does not occur freely, but rather faces many forms of hindrance, including that from membranes and intracellular organelles[7]. Therefore, many non-Gaussian diffusion models, including bi-exponential, stretched-exponential, and kurtosis models, have been developed to describe the complicated behavior of water diffusion. Some previous studies have suggested that the bi-exponential model [8, 9], stretched-exponential model [10], and diffusion kurtosis imaging [11] might provide useful information for PCa evaluation. However, the observed benefits from those non-Gaussian diffusion models required validation [12, 13], warranting further studies to explore and compare their roles in the diagnosis of PCa.

The choice of an appropriate b-value is another crucial aspect of DWI that affects PCa detection efficiency. The appropriate b-value is essential for calculating precise diffusion parameters to aid PCa diagnosis [14, 15]. Nevertheless, the maximum b-values used in prostate studies have varied greatly, from 500 to 3000 s/mm^2^[12, 16, 17]. while the brain delivered values up to 5000 s/mm^2^[18]. An earlier study investigated the influence of b-value range on DWI parameters and its effect on diagnostic performance for PCa [16]. However, the maximum b-value in that study was 2300 s/mm^2^; the effect of an ultrahigh b-value (up to 4500 s/mm^2^) remains unclear.

In this study, we aimed to address the influence of the range of b-values on the discriminatory value of the diffusion parameters of four different mathematical models (mono-exponential, bi-exponential, stretched-exponential and kurtosis) and to determine the optimal parameter and its optimal b-value range for PCa detection.

## Materials and methods

### Patients

This prospective study was approved by the Tongji Hospital, Tongji Medical College, Huazhong University of Science and Technology Institutional Review Board; written informed consent was obtained from each patient prior to examination. Between May 2014 and March 2015, patients with clinically suspected PCa due to either a rising or elevated prostate-specific antigen (PSA) level and/or palpable prostatic nodule, with no prior therapy, were recruited to this study. Initially, 166 consecutive patients were identified. Patients were excluded if a) they had histological confirmation of no cancer (n = 43); b) they had no histological confirmation of cancer (n = 51); c) the images were of poor quality due to susceptibility artifact or movement (n = 4); or d) the cancerous focus was located in the transition zone (n = 12). After these exclusions, 56 patients remained.

### MRI protocol

All examinations were performed on a 3.0 T GE 750 discovery system MRI scanner (General Electric, Milwaukee, WI, USA) with a 32–element torso phased-array coil. An endorectal coil was not used. The entire prostate gland and seminal vesicles were imaged in axial, sagittal and coronal slices, using a T2-weighted fast-recovery fast spin echo sequence with the following parameters TR range/TE range, 3322-3849/108-118 ms; slice thickness, 3 mm; inter-slice gap, 0 mm; FOV, 180×180 mm^2^; and matrix, 320×256. Transversal T1-weighted images were acquired with the following parameters: TR, 446 ms; TE, 8.1 ms; slice thickness, 5 mm; inter-slice gap, 0 mm; FOV, 360×360 mm^2^; and matrix, 320×224.

Axial DWI was acquired using a single-shot echo-planar imaging pulse sequence with the following parameters: TR, 2500 ms; TE, 84.1 ms; slice thickness, 5 mm; inter-slice gap, 0 mm; FOV, 400×280 mm^2^; matrix, 128×96, reconstruction matrix size 256 × 256; bandwidth, 250 Hz/pixel; acceleration factor of 2; and 21 b values (number of excitations) 0 (1), 20 (1), 50 (1), 80 (1), 100 (1), 150 (1), 200 (1), 400 (2), 600 (2), 800 (2), 1000 (4), 1200 (4), 1500 (4), 1800 (4), 2000 (6), 2400 (6), 2800 (6), 3200 (8), 3600 (8), 4000 (10), and 4500 (10) s/mm^2^. The DWI parameter was optimized to ensure a sufficient SNR at the maximum b-values up to 4500mm/s^2^. The total acquisition time was 10 minutes and 20 seconds. An additional 5mm section thickness axial T2-weighted image was scanned to match the DWI to provide anatomical information.

### TRUS-guided biopsy

After the MRI examination, all patients underwent transrectal ultrasound (TRUS)-guided biopsies. All the biopsies were performed by a single urologist with more than 20 years of experience using an ultrasound system (Hawk 2102, BK Medical, Denmark) equipped with a 5.1 MHz endocavitary probe, with a spring-loaded biopsy gun with an 18 gauge core biopsy needle. To match biopsy sextants and MR images, the PZ of the prostate was divided into six regions in MRI, which corresponded to the TRUS-guided biopsy (apex, mid gland, and base on each side of the PZ), and two core biopsies per region were obtained. Cores were individually labeled according to their location with respect to the biopsy scheme. Each specimen was histologically analyzed as cancerous or noncancerous by an experienced pathologist (with > 15 years of experience).

### MRI data assessment

MRI assessment was performed according to the methods used by Rosenkrantz *et al*. and Li *et al*.[11, 19], and post-processing was performed using the FUNCTOOL (GE Healthcare 4.6) workstation. MR images were interpreted by the consensus of two observers (ZY Feng, and XD Min) with more than 5 years of experience in prostate MRI (each observer had read more than one thousand prostate MRIs). Disagreements between the two observers were resolved by a re-evaluation with the study coordinator, a radiologist with more than 15 years of experience in prostate MRI. On the MR images, the prostate was divided into approximately equal thirds in the cranio-caudal direction, with each third demarcating the left and right basal, mid, and apical sextants, corresponding to the TRUS-guided biopsy. The readers treated each sextant as a separate focus for purposes of the analysis. Regions of interest (ROIs) were placed within the proven cancerous tissues and benign PZs on the T2WI and then automatically copied to DWI. The focus suspicious for PCa with a diameter of not less than 5 mm was delineated on the MRI of the prostate corresponding to the biopsy sextants. If both biopsies obtained in one region were negative or positive for PCa, the region was considered cancer negative or cancer positive respectively. If one biopsy was positive for cancer but the other was negative, the region was still considered cancer positive [11]. The sizes of the ROIs were chosen to be as large as possible with minimal contamination from uninvolved tissues. The SNR for each ROI was calculated for DWI with b-values of 4500 s/mm^2^. The SNR was defined as the ratio between the average signal intensity of ROIs and the mean of the standard deviation (SD) of the signals in the bilateral internal obturator muscles[20].

### Modeling

**Fig 1:**
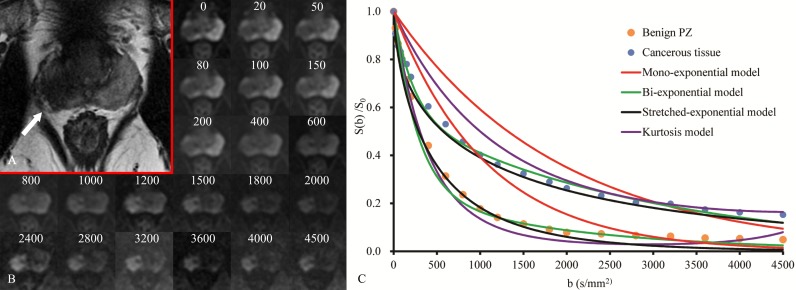
Prostate cancer in a 61-year-old patient with high serum PSA level of 19.43 ng/ml. (a)Transverse T2-weighted anatomical image shows a hypointense lesion (white arrow) in the right middle peripheral zone of the prostate; (b) A series of b-value images are shown with the corresponding location to the transverse T2-weighted (unites, s/mm^2^); (c) Measured signal and fitted curves of cancerous tissue and the opposite side benign tissue using maximum b-value of 4500 s/mm^2^ (group D).

All analyses in the current study were performed on an ROI level [13, 21]. We performed the non-linear least-square fitting based on the Levenberg-Marquardt algorithm using Matlab (MathWorks, Natick, MA, USA) to fit the following models to the mean signal of each ROI **(Fig 1)**:

1. Mono-exponential model
  $$S(b)/S_{0}=\exp(\text{-}b\cdot ADC)$$ (1)
2. Bi-exponential model
  $$S(b)/S_{0}=f\cdot \exp(\text{-}b\cdot D*)+(1\;\text{-}\;f)\cdot \exp(\;\text{-}b\cdot <D>)$$ (2)
3. Stretched exponential model
  $$S(b)/S_{0}=\exp[\text{-}{\left(b\cdot DDC\right)}^{\alpha }]$$ (3)
4. Diffusion kurtosis imaging
  $$S(b)/S_{0}=\exp(\text{-}b\cdot D_{app}+b^{2}\cdot D_{app}{}^{2}\cdot K_{app}/6)$$ (4)

Where S(b) is the signal intensity at a particular b-value, and S_0_ is the signal intensity at b = 0 s/mm^2^. ADC is the diffusion coefficient of the mono-exponential model. <D>, D*, and f are the diffusion parameters of the bi-exponential model: f is the perfusion fraction, <D> is the pure molecular diffusion, and D* is the pseudo-diffusion coefficient. DDC is the diffusion coefficient of the stretched exponential model, and α is the water diffusion heterogeneity index between 0 and 1. D_app_ is the diffusion coefficient of the kurtosis model, and K_app_ is the kurtosis.

### Statistical analysis

To validate the dependence of the models on b-values, the b-values were divided into four different ranges: 0–1000, 0–2000, 0–3200, and 0–4500 s/mm^2^, grouped as A, B, C, and D, respectively.

To measure the goodness-of-fit, adjusted coefficients of determination (R^2^) for the groups were calculated among different models using Eqs (1)–(4). One-way ANOVA statistics and Bonferroni test were used for multiple comparisons of adjusted R^2^ between models.

ADC, <D>, D*, f, DDC, α, D_app_, and K_app_ were estimated in the benign PZs and cancerous tissues. The parameters were calculated using all the b-values in groups A, B, C, and D. Normal distribution was assessed using the Kolmogorov-Smirnov test; if the data presented a normal distribution, an independent *t*-test was performed to compare the differences in parameters between benign PZs and cancerous tissues. For non-normally distributed variables, the Mann-Whitney U test was used. Next, receiver operating characteristic curve (ROC) analysis was performed, and the area under the curve (AUC) was calculated to evaluate and compare the diagnostic accuracy of the parameters in distinguishing cancerous tissues from benign PZs. The diagnostic performance was expressed as the AUC. Differences in AUCs were assessed using the method of DeLong *et al*[22].

The adjusted R^2^ was calculated with Matlab (MathWorks, Natick, MA, USA). The statistical analysis was performed using SPSS software (SPSS for Windows 19.0, Chicago, IL, USA). The ROC analysis was performed using MedCalc version 13.0.0.0 for Windows (MedCalc Software, Mariakerke, Belgium). For all the significance level was set at *p*< 0.05.

## Results

The final study population comprised 56 PZ PCa patients (age range, 52–82 years; median age, 67 years). The median PSA level in these 56 patients was 26.036 ng/mL (range, 2.06–1000 ng/ml). Among the total 336 ROIs, 198 ROIs were evaluated as benign PZs, and 138 ROIs were evaluated as cancerous tissues. The SNR of the ROIs at the highest b-value of 4500 s/mm^2^was between 8.784 and 64.118. The mean (±SD) ROI SNRs in cancerous tissues and benign PZs were 27.784±10.245 and 17.035±8.107, respectively. The mean (±SD) ROI sizes in cancerous tissues and benign PZs were 169.780±107.914mm^2^ and 97.57±22.041mm^2^, respectively.

### Goodness-of-fit of the models

**Fig 2:**
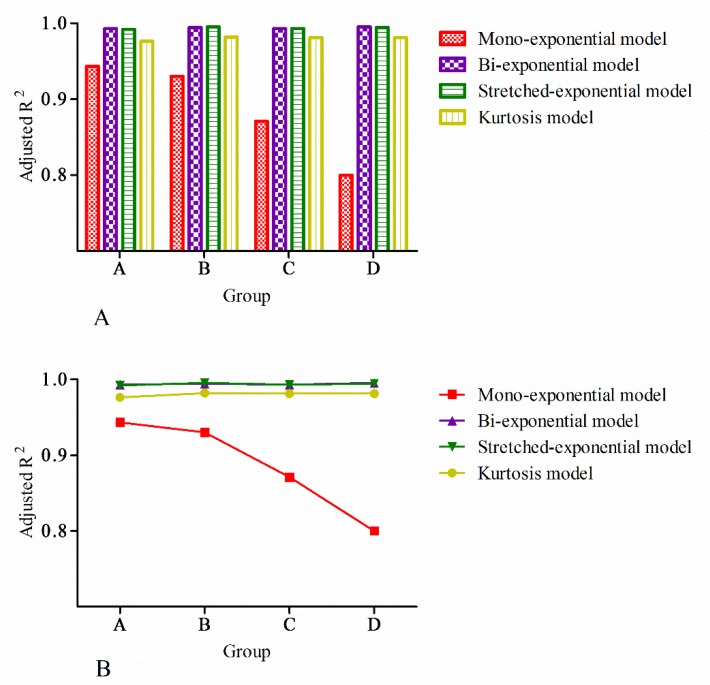
Graph showing variations in the adjusted R^2^ values of the mono-exponential, bi-exponential, stretched-exponential and kurtosis models with an increase in b-value. The mean value of the adjusted R^2^ of the mono-exponential model decreased with the increase of b-value, while the mean value of the adjusted R^2^ of the bi-exponential, stretched-exponential and kurtosis models were stable and excellent with the increase of b-value (It should be noted that, because the adjusted R^2^ values of the bi-exponential and stretched-exponential models were close, their curves have been superimposed).

The adjusted R^2^ was calculated to test the goodness-of-fit of the models. The mean values and standard deviations of the adjusted R^2^ are provided in **Table 1**. The bi-exponential and the stretched-exponential models provided the highest adjusted R^2^ among the four models in every group. There were no significant differences between the bi-exponential and stretched-exponential models in any of the four groups (*p*>0.05 for all). The goodness-of-fit of the kurtosis model was slightly less than that of the bi-exponential and the stretched-exponential models in all groups (*p*< 0.05 for all). The mono-exponential provided the worst goodness-of-fit in the four groups. Significant differences were observed between kurtosis and the other three models in all groups (*p*< 0.05 for all). Similarly, the mono-exponential model showed differences from the other three models in all groups (*p*< 0.05 for all). The mean value of the adjusted R^2^ of the mono-exponential model decreased with increasing b-value. No clear tendency could be established for the bi-exponential, stretched-exponential or kurtosis models (**Fig 2**)**.**

**Table 1:** Mean values and standard deviation of the adjusted R^2^.

Groups	Mono-exponential	Bi-exponential	Stretched-exponential	Kurtosis
A	0.943±0.077	0.993±0.032	0.992±0.037	0.976±0.045
B	0.930±0.040	0.994±0.016	0.995±0.019	0.982±0.029
C	0.871±0.050	0.993±0.020	0.993±0.022	0.981±0.036
D	0.800±0.084	0.995±0.008	0.994±0.010	0.981±0.022

### Summary of DWI parameters

**Fig 3:**
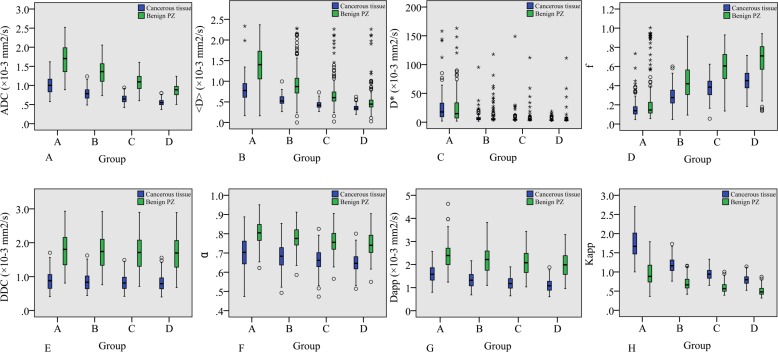
**Boxplot of diffusion parameters calculated using different b-value ranges (groups A, B, C and D)**. (a-b, d-g), the mean values of ADC, <D>, f, DDC, α, and D_app_ were significantly lower in cancerous tissues than in benign PZs in each group (h). The K_app_ in cancerous tissues was significantly higher than in benign PZs in each group. The value of D*, which had a large standard deviation, showed no significant difference between cancerous tissues and benign PZs in groups A and D but was significantly different in groups B and C(c).

The mean values and standard deviations of the diffusion parameters measured in groups A, B, C, and D are summarized in **Table 2**. The mean values of ADC, <D>, f, DDC, α, and D_app_ were found to be significantly lower in cancerous tissues than in benign PZs in each group (*p*< 0.001 for all). Similarly, the K_app_ in cancerous tissues was significantly higher than that in benign PZs in each group (*p*< 0.001 for all). We also found a significant difference in D* between cancerous tissues and benign PZs in groups B and C (*p<* 0.05), but not in groups A and D (*p* > 0.05). However, the D* showed a large standard deviation in all groups(**Fig 3)**.

**Table 2:** Diffusion parameters in benign PZs and cancerous tissues.

Parameter		Benign PZ	Cancerous tissue	*t/Z*	*p*
ADC	Group A	1.683±0.389	1.006±0.210	*Z* = -13.387	<0.001
	Group B	1.340±0.303	0.788±0.152	*Z* = -13.967	<0.001
	Group C	1.076±0.220	0.654±0.110	*Z* = -14.251	<0.001
	Group D	0.867±0.158	0.555±0.086	*Z* = -14.255	<0.001
<D>	Group A	1.397±0.467	0.793±0.271	*Z* = -11.879	<0.001
	Group B	0.956±0.385	0.543±0.117	*Z* = -12.668	<0.001
	Group C	0.683±0.328	0.428±0.085	*Z* = -11.120	<0.001
	Group D	0.506±0.305	0.353±0.070	*Z* = -8.147	<0.001
D*	Group A	24.076±26.215	25.489±26.357	*Z* = -1.276	0.202
	Group B	8.055±12.797	7.899±9.045	*Z* = -3.452	0.001
	Group C	5.265±9.596	6.233±12.980	*Z* = -2.708	0.007
	Group D	4.445±9.269	4.081±2.764	*Z* = -1.405	0.160
f	Group A	0.235±0.226	0.161±0.097	*Z* = -2.045	0.041
	Group B	0.438±0.179	0.293±0.104	*Z* = -7.832	<0.001
	Group C	0.591±0.174	0.386±0.112	*Z* = -10.359	<0.001
	Group D	0.683±0.161	0.457±0.120	*Z* = -11.509	<0.001
DDC	Group A	1.780±0.510	0.884±0.281	*Z* = -13.542	<0.001
	Group B	1.736±0.503	0.858±0.252	*Z* = -13.703	<0.001
	Group C	1.709±0.506	0.832±0.230	*Z* = -13.799	<0.001
	Group D	1.693±0.510	0.818±0.234	*Z* = -13.767	<0.001
α	Group A	0.800±0.068	0.699±0.088	*t* = -11.414 (df = 245.122)	<0.001
	Group B	0.776±0.059	0.682±0.068	*t* = -13.464 (df = 334)	<0.001
	Group C	0.757±0.060	0.663±0.059	*t* = -14.223 (df = 334)	<0.001
	Group D	0.743±0.063	0.647±0.051	*t* = -15.535 (df = 327.863)	<0.001
D_app_	Group A	2.378±0.559	1.609±0.382	*t* = -14.984 (df = 333.896)	<0.001
	GroupB	2.201±0.544	1.335±0.316	*t* = -18.396 (df = 324.708)	<0.001
	Group C	2.086±0.535	1.189±0.272	*Z* = -13.360	<0.001
	Group D	1.991±0.529	1.097±0.260	*Z* = -13.525	<0.001
K_app_	Group A	0.967±0.309	1.744±0.387	*Z* = -13.730	<0.001
	Group B	0.715±0.171	1.185±0.214	*Z* = -14.185	<0.001
	Group C	0.597±0.131	0.948±0.150	*Z* = -14.249	<0.001
	Group D	0.505±0.116	0.801±0.126	*Z* = -14.126	<0.001

Mean ± SD of ADC, <D>, D*, DDC, and D_app;_ all values are in units of 10^−3^ mm^2^/s; f, α, and K_app_ for all groups (A, B, C and D), both in benign PZs and cancerous tissues are unitless.

### Analysis of ROC curves

**Fig 4:**
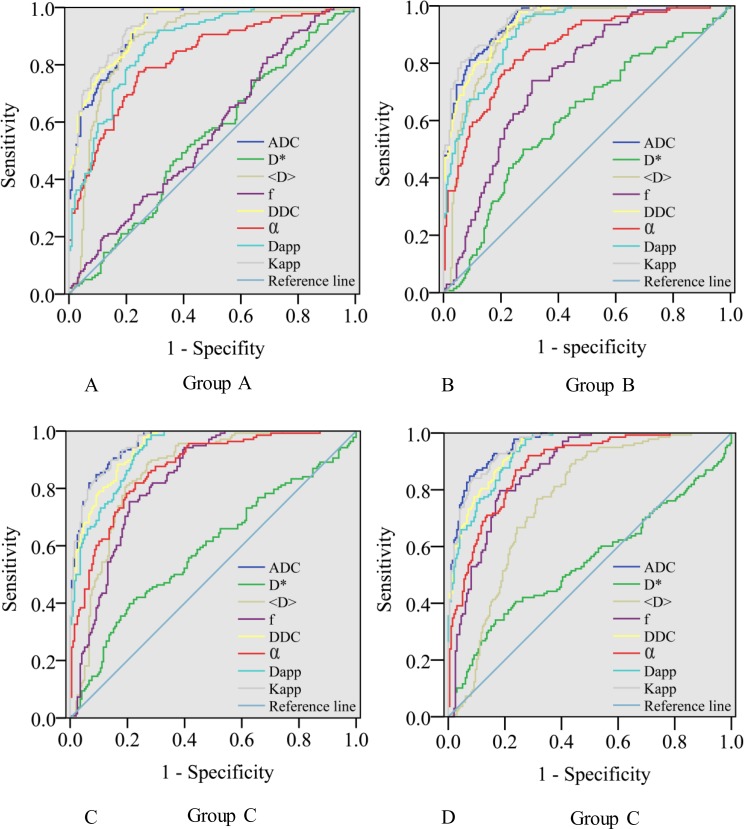
ROC curve analyses show the diagnostic accuracy of the diffusion parameters in distinguishing between cancerous tissues and benign PZs. In group A, K_app_ had the largest AUC (0.940), but the AUCs of ADC and K_app_ were not significantly different (*p* = 0.070). In groups B and C, the AUCs of ADC and K_app_ were comparable and significantly higher than those of the other parameters. In group D, the AUCs of ADC was slightly higher than that of K_app_ (0.957 vs 0.953, *p* = 0.002), and both were significantly higher than those of other parameters (*p*< 0.001 for all).

The AUC, cutoff, sensitivity, and specificity values of the parameters for distinguishing cancerous tissues from benign PZs are reported in **Table 3**. The ROC curves of each parameter calculated using different b-value ranges are shown in **Fig 4**.

**Table 3:** Diagnostic performance of parameters calculated with different b-value ranges.

		AUC	Cut-off	Sensitivity	Specificity	*p* value
ADC	Group-A	0.929±0.013	1.415	97.8	72.7	<0.001
	Group-B	0.948±0.010	1.126	98.6	74.2	<0.001
	Group-C	0.957±0.009	0.794	89.9	85.9	<0.001
	Group-D	0.957±0.009	0.649	84.8	92.4	<0.001
<D>	Group-A	0.881±0.020	1.010	88.4	79.3	<0.001
	Group-B	0.906±0.017	0.701	90.6	79.3	<0.001
	Group-C	0.856±0.021	0.533	89.1	72.7	<0.001
	Group-D	0.761±0.026	0.430	89.1	56.6	<0.001
D*	Group-A	0.541±0.032	17.346	51.4	58.6	0.197
	Group-B	0.611±0.031	5.998	50.0	72.2	<0.001
	Group-C	0.587±0.032	4.575	39.9	79.3	0.007
	Group-D	0.545±0.034	4.0339	34.1	83.3	0.178
f	Group-A	0.566±0.031	0.195	82.6	30.8	0.037
	Group-B	0.751±0.026	0.336	73.9	69.2	<0.001
	Group-C	0.832±0.022	0.447	75.4	79.3	<0.001
	Group-D	0.869±0.020	0.544	79.7	81.8	<0.001
DDC	Group-A	0.934±0.012	1.442	97.8	72.7	<0.001
	Group-B	0.939±0.011	1.369	97.8	73.7	<0.001
	Group-C	0.942±0.011	1.331	97.8	74.2	<0.001
	Group-D	0.941±0.011	1.318	97.8	74.2	<0.001
α	Group-A	0.821±0.023	0.762	77.5	75.8	<0.001
	Group-B	0.852±0.021	0.739	81.2	75.3	<0.001
	Group-C	0.870±0.020	0.716	81.9	77.3	<0.001
	Group-D	0.884±0.018	0.709	92.0	71.7	<0.001
D_app_	Group-A	0.872±0.019	2.099	92.0	68.7	<0.001
	Group-B	0.911±0.015	1.871	95.7	72.7	<0.001
	Group-C	0.928±0.013	1.728	97.1	73.2	<0.001
	Group-D	0.934±0.012	1.630	97.8	72.7	<0.001
K_app_	Group-A	0.940±0.011	1.251	92.8	79.8	<0.001
	Group-B	0.955±0.009	0.966	85.5	88.9	<0.001
	Group-C	0.957±0.009	0.770	89.9	85.9	<0.001
	Group-D	0.953±0.010	0.676	84.1	91.4	<0.001

The cut-off values of ADC, <D>, D*, DDC, and D_app,_ are in units of 10^−3^ mm^2^/s.

The parameters ADC, <D>, DDC and K_app_ provided the highest AUCs among the four models. The AUCs of ADC in groups C and D were comparable (p = 0.980) and were significantly higher than those of groups A and B (*p*< 0.05 for all). The AUCs of <D> calculated in groups A, B, and C were significantly higher than in group D (*p*< 0.001 for all). The AUC of <D> in groups A and B showed no significant difference (*p* = 0.136), and the value of group B was significantly higher than in groups C and D (*p*< 0.001 for all). The AUCs of DDC calculated using groups B, C and D showed no significant difference (*p* > 0.05 for all). The AUCs of DDC calculated using groups B and D were significantly higher than in group A (*p* = 0.016 and *p* = 0.014, respectively). The AUCs of DDC calculated using groups A and C showed no significant difference (*p* = 0.102). The AUCs of K_app_ calculated using groups B, C, D were significantly higher than in group A (*p*< 0.05 for all); however, no significant difference was observed among groups B, C, and D (*p* > 0.05 for all).

For the parameters calculated using the b-value of group A, the AUCs of DDC and K_app_ were not significantly different (*p* = 0.204); the AUCs of ADC and K_app_ were not significantly different (*p* = 0.070); the AUC of DDC was slightly higher than the AUC of ADC (*p* = 0.014); and the AUCs of ADC, DDC and K_app_ were significantly higher than those of the other parameters (*p*< 0.05 for all). For the parameters calculated using the b-values of groups B and C, the AUCs of ADC and K_app_ were similar (*p* = 0.07 and *p* = 0.954, respectively), but were significantly higher than those of the other parameters (*p*< 0.001 for all). For the parameters calculated using the b-value of group D, the AUC of ADC was slightly higher than that of K_app_ (0.957 vs 0.953, *p* = 0.002); the AUCs of ADC and K_app_ were significantly higher than those of the other parameters (*p*< 0.001 for all).

## Discussion

Our study demonstrated that for PZ PCa detection, the parameters derived from the bi-exponential, stretched-exponential, and kurtosis models did not reveal significantly superior diagnostic performance relative to conventional ADC. ADC derived from conventional mono-exponential high b-value (3200 s/mm^2^) provided excellent diagnostic performance in PZ PCa detection, such that no extra benefit was obtained from cumbersome non-Gaussian models (bi-exponential, stretched-exponential, and kurtosis models) and time-consuming over-high b-value (> 3200 s/mm^2^).

Appropriate b-values must be carefully selected for the use of DWI in PCa detection. In clinical applications, DWI is typical performed using b-values 800–1500 s/mm^2^[4, 23]. Nevertheless, the clinical benefits of high-b-value DWI have been explored from head to body[17, 24, 25]. Until now, consensus on the optimal b-value range of DWI for PCa diagnosis has been lacking. As reported in a previous study, b-value distribution mainly influences the repeatability of DWI-derived parameters rather than the diagnostic performance [26]; however, the influence of b-value distribution on diagnostic performance is beyond the scope of this study. Our study focused on the influence of b-value range on the diagnostic performance of the parameters of the various DWI models.

Our study showed that, for PCa detection, the diagnostic performance of DDC and K_app_ calculated using b-values up to 2000 s/mm^2^ outperformed those calculated using b-values up to a maximum of 1000 s/mm^2^. According to a previous study, DWI with a maximum b-value of 2000 s/mm^2^ is beneficial for the estimation of the stretched-exponential and kurtosis model parameters[27]. Our results are also in agreement with a study by Mazzoni[16], which found that the K_app_ demonstrated higher AUC for PCa detection using a higher b-value (2300 s/mm^2^) than a low b-value (800 s/mm^2^). However, the highest b-value they used was only 2300 s/mm^2^. For brain kurtosis imaging, empirical evidence indicates that maximum b-values of 2000 to 3000 s/mm^2^ are appropriate[7, 28]. However, no reasonable maximum b-value has been recommended for prostate stretched-exponential and kurtosis imaging. Our results revealed that higher b-values above 2000 s/mm^2^ provided no extra diagnostic value. Therefore, maximum b-values of approximately 2000 s/mm^2^ may be appropriate for prostate stretched-exponential and kurtosis model imaging. The suggestion of Rosenkrantz *et al*., that a maximal b-value of 1500–2000 s/mm^2^ is suitable for body kurtosis imaging, also supports this conclusion[28]. For the bi-exponential model, <D> provided the highest diagnostic value. The AUC of <D> was comparable in group A and group B and was significantly decreased in group C and D. This phenomenon reminds us to pay attention to the maximum b-value of the bi-exponential model. Some studies showed that the parameters of the bi-exponential model depended heavily on the choice of b-values and that it is critical to select an appropriate range of b-values in prostate imaging to obtain accurate parameters[15, 29]. In contrast to <D>, DDC and K_app_, the diagnostic value of ADC calculated using a maximum b-value of 3200 s/mm^2^ was superior to those derived using maximum b-values of 2000 s/mm^2^ and 1000 s/mm^2^. The increased maximum b-value provided greater contrast between cancerous and non-cancerous tissues, adding to the diagnostic performance. However, when the b-value was higher than 3200 s/mm^2^_,_ the rapid decrease in the goodness-of-fit (0.871 to 0.800) may outweigh the extra benefit of ADC.

Non-Gaussian diffusion models have been proposed to describe complicated water diffusion behavior and are believed to provide a more accurate description of the DWI signal decay curves obtained using higher b-values [30]. Our results showed that the diagnostic performance of parameters derived from a bi-exponential model was low despite excellent goodness-of-fit. The better fit of the bi-exponential model may result from the model addressing a large number of free parameters. However, the additional parameters may cause over-fitting of the data, resulting in poor repeatability and reliability [12, 13, 30, 31]. A study by Merisaari *et al*. found that, compared with the mono-exponential, stretched-exponential, and kurtosis models, the parameters of the bi-exponential model demonstrated the worst repeatability and diagnostic performance [26]. The poor repeatability and reliability of the bi-exponential model limits its clinical utility. When calculated with a high b-value (≥2000 mm^2^/s), the ROC analysis in our study did not reveal significantly superior performance of the parameters derived from the stretched-exponential and kurtosis models over the conventional ADC for PCa detection, although a higher-quality fit to DWI signal decay was provided. These results are consistent with several previous studies [13, 32, 33]. High reliability and repeatability are preconditions for using parameters derived from quantitative diffusion models for disease diagnosis and characterization, and this may be why the mono-exponential model is superior at maintaining robust diagnostic performance. Nevertheless, the additional value for PCa of non-Gaussian diffusion models compared with the mono-exponential model remains controversial and requires further research. It is well known that the mono-exponential, bi-exponential, stretched-exponential, and kurtosis models all belong to phenomenological DWI signal models. However, until now, their physiologic basis remained unclear, necessitating further evaluation of the physiologic basis of DWI[34].

There were several limitations in this study. First, this study took the TRUS-guided biopsy as the standard of reference. The use of whole-mount histopathology would improve the accuracy of the agreement between MR images and histopathology. However, it is unreasonable to expect all of our subjects to undergo prostatectomy. Because TRUS-guided biopsy has good specificity but poor sensitivity, a small tumor focus may be missed, resulting in a false-negative diagnosis. In our study, we focused primarily on large tumors, which could partially obviate this problem. Moreover, TRUS-guided biopsy has been the standard of reference in a large number of studies [11, 19, 35, 36], which also illustrated the feasibility of the method used in our study. Second, the role of DWI parameters in the assessment of the transition zone was not evaluated, because TRUS-guided biopsy may miss some cancers that arise in the transition zone and because 70–75% of PCa are located in the PZ. Additionally, DWI is the dominant sequence for PZ PCa detection, while the dominant sequence for transition zone PCa is T2WI[5]; thus, it is more important to evaluate the diagnostic performance of DWI for PZ PCa. Third, compared with whole mount step-section histopathology, TRUS-guided biopsy may lead to the under-grading of a fraction of cancers, therefore this study did not aim to explore the predictive accuracy of different mathematical models with regard to Gleason score. Fourth, the influence of SNR on diffusion model parameters of different b-value ranges was not evaluated. The issue of low SNR must be considered for diffusion imaging, especially for high-b-value images. Although high b-values up to 4500s/mm^2^ were used in our study, the SNR of the highest images still reached an extremely high level. The following strategies may have played a positive role in increasing SNR. First, we included imaging at a higher field strength (3.0 Tesla). Second, we increased the number of excitations (10 for the highest b-value image). Third, we used a slice thickness of 5 mm, which is thicker than the 3mm commonly used in clinical settings. Fourth, the resolution we used was 3.125×2.917mm rather than 2.5×2.5mm, which is routinely used in clinical settings. Despite the limitations, we believe our methodical strategies provide sufficient validity for the principal results of our study.

## Conclusion

In conclusion, our study suggested that ADC derived from a conventional mono-exponential model using a high b-value (3200s /mm^2^) DWI is an optimal choice for clinical routine application in PZ PCa detection. For PCa detection, the diagnostic value of parameters derived from non-mono-exponential diffusion models or higher b-values (> 3200 s /mm^2^) warrant further multicenter exploration. Furthermore, as highly parameterized non-Gaussian diffusion models have a higher information content than the single-parameter mono-exponential model, they may provide useful information for differentiating PCa from other prostatic lesions and might be promising for monitoring PCa progression or response to therapy, which also needs further evaluation.

## Supporting information

S1 Data Computed parameters for benign and cancerous tissues. (XLS)

S1 Table Multiple comparisons for each parameters in Table 2. (DOC)

S2 Table Multiple comparisons for AUC in Table 3. (DOC)
