# Correction: Evaluation of different mathematical models and different b-value ranges of diffusion-weighted imaging in peripheral zone prostate cancer detection using b-value up to 4500 s/mm^2^

**DOI:** 10.1371/journal.pone.0184609

**Published:** 2017-09-06

**Authors:** The PLOS ONE Staff

Xin Yang is not included in the author byline. Dr. Yang should be listed as the sixth author and affiliated with the School of Electronic Information and Communications, Huazhong University of Science and Technology, Wuhan, Hubei, China. The publisher apologizes for the error.

The correct citation is: Feng Z, Min X, Margolis DJA, Duan C, Chen Y, Yang X, et al. (2017) Evaluation of different mathematical models and different b-value ranges of diffusion-weighted imaging in peripheral zone prostate cancer detection using b-value up to 4500 s/mm^2^. PLoS ONE 12(2): e0172127. https://doi.org/10.1371/journal.pone.0172127

The following information is missing from the Funding section: This work was supported by the National Natural Science Foundation of China (grant nos. 81171307, 81671656).

## References

[1] Feng Z, Min X, Margolis DJA, Duan C, Chen Y, Sah VK, et al (2017) Evaluation of different mathematical models and different b-value ranges of diffusion-weighted imaging in peripheral zone prostate cancer detection using b-value up to 4500 s/mm^2^. PLoS ONE 12(2): e0172127 https://doi.org/10.1371/journal.pone.0172127 28199367 10.1371/journal.pone.0172127 PMC5310778

