# Supplementary material for: Evaluation of different mathematical models and different b-value ranges of diffusion-weighted imaging in peripheral zone prostate cancer detection using b-value up to 4500 s/mm2
Source: PLoS One. 2017 Feb 15;12(2):e0172127. doi: 10.1371/journal.pone.0172127 (PMC5310778; doi:10.1371/journal.pone.0172127)
Supplement: S1 Table — (DOC) [file pone.0172127.s002.doc]

**S2 Table. Multiple comparisons for each parameters in Table 2**

For the normally distributed data, we used One-way ANOVA statistics and Bonferroni correction for multiple comparisons. For the data that were not normally distributed, we replaced the One-way ANOVA statistics and Bonferroni correction with the nonparametric-Wilcoxon test.

Table 1. Multiple comparisons for ADC of normal PZs

| Groups B | Z=-12.201  P < 0.001 |  |  |
| --- | --- | --- | --- |
| Groups C | Z=-12.201  P < 0.001 | Z=-12.201  P < 0.001 |  |
| Groups D | Z=-12.201  P < 0.001 | Z=-12.201  P < 0.001 | Z=-12.201  P < 0.001 |
|  | Groups A | Groups B | Groups C |

Table 2. Multiple comparisons for ADC of cancerous tissues

| Groups B | Z=-10.192  P < 0.001 |  |  |
| --- | --- | --- | --- |
| Groups C | Z=-10.090  P < 0.001 | Z=-9.709  P < 0.001 |  |
| Groups D | Z=-10.192  P < 0.001 | Z=-10.192  P < 0.001 | Z=-9.357  P < 0.001 |
|  | Groups A | Groups B | Groups C |

Table 3. Multiple comparisons for <D> of normal PZs

| Groups B | Z=-11.834  P < 0.001 |  |  |
| --- | --- | --- | --- |
| Groups C | Z=-12.111  P < 0.001 | Z=-12.170  P < 0.001 |  |
| Groups D | Z=-12.157  P < 0.001 | Z=-12.189  P < 0.001 | Z=-12.195  P < 0.001 |
|  | Groups A | Groups B | Groups C |

Table 4. Multiple comparisons for <D> of cancerous tissues

| Groups B | Z=-9.824  P < 0.001 |  |  |
| --- | --- | --- | --- |
| Groups C | Z=-10.018  P < 0.001 | Z=-9.165  P < 0.001 |  |
| Groups D | Z=-10.132  P < 0.001 | Z=-10.120  P < 0.001 | Z=-9.195  P < 0.001 |
|  | Groups A | Groups B | Groups C |

Table 5. Multiple comparisons for D* of normal PZs

| Groups B | Z=-11.873  P < 0.001 |  |  |
| --- | --- | --- | --- |
| Groups C | Z=-12.008  P < 0.001 | Z=-12.190  P < 0.001 |  |
| Groups D | Z=-12.059  P < 0.001 | Z=-12.198  P < 0.001 | Z=-12.200  P < 0.001 |
|  | Groups A | Groups B | Groups C |

Table 6. Multiple comparisons for D* of cancerous tissues

| Groups B | Z=-9.512  P < 0.001 |  |  |
| --- | --- | --- | --- |
| Groups C | Z=-9.233  P < 0.001 | Z=-8.179  P < 0.001 |  |
| Groups D | Z=-10.111  P < 0.001 | Z=-10.177  P < 0.001 | Z=-7.299  P < 0.001 |
|  | Groups A | Groups B | Groups C |

Table 7. Multiple comparisons for f of normal PZs

| Groups B | Z=-9.990  P < 0.001 |  |  |
| --- | --- | --- | --- |
| Groups C | Z=-11.561  P < 0.001 | Z=-12.179  P < 0.001 |  |
| Groups D | Z=-11.924  P < 0.001 | Z=-12.194  P < 0.001 | Z=-12.196  P < 0.001 |
|  | Groups A | Groups B | Groups C |

Table 8. Multiple comparisons for f of cancerous tissues

| Groups B | Z=-9.484  P < 0.001 |  |  |
| --- | --- | --- | --- |
| Groups C | Z=-9.669  P < 0.001 | Z=-8.326  P < 0.001 |  |
| Groups D | Z=-10.022  P < 0.001 | Z=-10.141  P < 0.001 | Z=-7.408  P < 0.001 |
|  | Groups A | Groups B | Groups C |

Table 9. Multiple comparisons for DDC of normal PZs

| Groups B | Z=-11.808  P < 0.001 |  |  |
| --- | --- | --- | --- |
| Groups C | Z=-12.158  P < 0.001 | Z=-12.201  P < 0.001 |  |
| Groups D | Z=-12.198  P < 0.001 | Z=-12.201  P < 0.001 | Z=-12.201  P < 0.001 |
|  | Groups A | Groups B | Groups C |

Table 10. Multiple comparisons for DDC of cancerous tissues

| Groups B | Z=-5.841  P < 0.001 |  |  |
| --- | --- | --- | --- |
| Groups C | Z=-5.295  P < 0.001 | Z=-5.076  P < 0.001 |  |
| Groups D | Z=-7.544  P < 0.001 | Z=-10.192  P < 0.001 | Z=-6.674  P < 0.001 |
|  | Groups A | Groups B | Groups C |

Table 11. Multiple comparisons for α of normal PZs

| Groups B | P = 0.001 |  |  |
| --- | --- | --- | --- |
| Groups C | P < 0.001 | P =0.017 |  |
| Groups D | P < 0.001 | P < 0.001 | P < 0.207 |
|  | Groups A | Groups B | Groups C |

Table 12. Multiple comparisons for α of cancerous tissues

| Groups B | P =0.247 |  |  |
| --- | --- | --- | --- |
| Groups C | P < 0.001 | P = 0.1231 |  |
| Groups D | P < 0.001 | P < 0.001 | P = 0.274 |
|  | Groups A | Groups B | Groups C |

Table 13. Multiple comparisons for D_app_ of normal PZs

| Groups B | Z=-12.102  P < 0.001 |  |  |
| --- | --- | --- | --- |
| Groups C | Z=-12.180  P < 0.001 | Z=-12.201  P < 0.001 |  |
| Groups D | Z=-12.201  P < 0.001 | Z=-12.201  P < 0.001 | Z=-12.201  P < 0.001 |
|  | Groups A | Groups B | Groups C |

Table 14. Multiple comparisons for D_app_ of cancerous tissues

| Groups B | Z=-10.192  P < 0.001 |  |  |
| --- | --- | --- | --- |
| Groups C | Z=-9.888  P < 0.001 | Z=-8.547  P < 0.001 |  |
| Groups D | Z=-10.192  P < 0.001 | Z=-10.192  P < 0.001 | Z=-8.107  P < 0.001 |
|  | Groups A | Groups B | Groups C |

Table15. Multiple comparisons for K_app_ of normal PZs

| Groups B | Z=-12.064  P < 0.001 |  |  |
| --- | --- | --- | --- |
| Groups C | Z=-12.169  P < 0.001 | Z=-12.200  P < 0.001 |  |
| Groups D | Z=-12.198  P < 0.001 | Z=-12.201  P < 0.001 | Z=-12.201  P < 0.001 |
|  | Groups A | Groups B | Groups C |

Table16. Multiple comparisons for K_app_ of cancerous tissues

| Groups B | Z=-10.192  P < 0.001 |  |  |
| --- | --- | --- | --- |
| Groups C | Z=-10.185  P < 0.001 | Z=-9.531  P < 0.001 |  |
| Groups D | Z=-10.192  P < 0.001 | Z=-10.192  P < 0.001 | Z=-9.801  P < 0.001 |
|  | Groups A | Groups B | Groups C |
