# Supplementary material for: Evaluation of different mathematical models and different b-value ranges of diffusion-weighted imaging in peripheral zone prostate cancer detection using b-value up to 4500 s/mm2
Source: PLoS One. 2017 Feb 15;12(2):e0172127. doi: 10.1371/journal.pone.0172127 (PMC5310778; doi:10.1371/journal.pone.0172127)
Supplement: S2 Table — (DOC) [file pone.0172127.s003.doc]

**S3 Table. Multiple comparisons for AUC in Table 3**

Table 1. Multiple comparisons for AUC of ADC

| Groups B | Z=5.327  P < 0.001 |  |  |
| --- | --- | --- | --- |
| Groups C | Z=4.229  P < 0.001 | Z=2.090  P = 0.037 |  |
| Groups D | Z=4.789  P < 0.001 | Z=2.917  P = 0.004 | Z=0.025  P=0.980 |
|  | Groups A | Groups B | Groups C |

Table 2. Multiple comparisons for AUC of <D>

| Groups B | Z=1.491  P = 0.136 |  |  |
| --- | --- | --- | --- |
| Groups C | Z=1.081  P = 0.280 | Z=3.836  P < 0.001 |  |
| Groups D | Z=4.334  P < 0.001 | Z=7.133  P < 0.001 | Z=5.912  P < 0.001 |
|  | Groups A | Groups B | Groups C |

Table 3. Multiple comparisons for AUC of D*

| Groups B | Z=2.587  P = 0.010 |  |  |
| --- | --- | --- | --- |
| Groups C | Z=1.299  P = 0.194 | Z=0.970  P = 0.332 |  |
| Groups D | Z=0.122  P < 0.001 | Z=3.245  P = 0.001 | Z=1.794  P = 0.073 |
|  | Groups A | Groups B | Groups C |

Table 4. Multiple comparisons for AUC of f

| Groups B | Z=5.903  P < 0.001 |  |  |
| --- | --- | --- | --- |
| Groups C | Z=7.68  P < 0.001 | Z=4.916  P < 0.001 |  |
| Groups D | Z=9.030  P < 0.001 | Z=7.052  P < 0.001 | Z=3.388  P < 0.001 |
|  | Groups A | Groups B | Groups C |

Table 5. Multiple comparisons for AUC of DDC

| Groups B | Z=2.408  P = 0.016 |  |  |
| --- | --- | --- | --- |
| Groups C | Z=1.636  P =0.102 | Z=0.713  P = 0.476 |  |
| Groups D | Z=2.454  P = 0.014 | Z=1.743  P = 0.081 | Z=0.241  P = 0.810 |
|  | Groups A | Groups B | Groups C |

Table 6. Multiple comparisons for AUC of α

| Groups B | Z=3.369  P < 0.001 |  |  |
| --- | --- | --- | --- |
| Groups C | Z=3.304  P = 0.001 | Z=1.885  P = 0.060 |  |
| Groups D | Z=4.905  P < 0.001 | Z=4.824  P < 0.001 | Z=1.720  P =0.086 |
|  | Groups A | Groups B | Groups C |

Table 7. Multiple comparisons for AUC of D_app_

| Groups B | Z=5.669  P < 0.001 |  |  |
| --- | --- | --- | --- |
| Groups C | Z=5.168  P < 0.001 | Z=2.864  P = 0.004 |  |
| Groups D | Z=6.225  P < 0.001 | Z=5.878  P < 0.001 | Z=1.123  P =0.261 |
|  | Groups A | Groups B | Groups C |

Table 8. Multiple comparisons for AUC of K_app_

| Groups B | Z=3.175  P = 0.002 |  |  |
| --- | --- | --- | --- |
| Groups C | Z=2.574  P = 0.010 | Z=0.436  P =0.663 |  |
| Groups D | Z=2.409  P =0.016 | Z=0.538  P =0.591 | Z=0.909  P = 0.364 |
|  | Groups A | Groups B | Groups C |
